# Whole-Genome Sequences of Two Listeria monocytogenes Serovar 1/2a Strains Responsible for a Severe Listeriosis Outbreak in Central Italy

**DOI:** 10.1128/genomeA.00236-18

**Published:** 2018-06-14

**Authors:** Massimiliano Orsini, Alessandra Cornacchia, Claudio Patavino, Marina Torresi, Patrizia Centorame, Vicdalia Aniela Acciari, Anna Ruolo, Maurilia Marcacci, Massimo Ancora, Marco Di Domenico, Iolanda Mangone, Giuliana Blasi, Anna Duranti, Cesare Cammà, Francesco Pomilio, Giacomo Migliorati

**Affiliations:** a Istituto Zooprofilattico Sperimentale dell’Abruzzo e del Molise, Teramo, Italy; b Istituto Zooprofilattico Sperimentale dell’Umbria e delle Marche, Perugia, Italy

## Abstract

We report the whole-genome sequences of two Listeria monocytogenes strains responsible for a severe invasive listeriosis outbreak in central Italy that occurred in 2015 and 2016. These two strains differ by a single band in their pulsed-field gel electrophoresis (PFGE) profiles.

## GENOME ANNOUNCEMENT

Listeria monocytogenes is a foodborne bacterial pathogen (1). Immunocompromised patients, the elderly, pregnant women, and newborns are at higher risk of developing invasive listeriosis, with high fatality rates (2). In central Italy in the period from January 2015 to February 2016, an increase in the notification of human listeriosis cases was reported (3), and we collected strains from patients and environmental samples; six patients died, and for all of them, listeriosis was confirmed as the major cause of death. Most of the strains isolated from clinical specimens exhibited two similar and common pulsed-field gel electrophoresis (PFGE) patterns in Europe, AscI.0183 ApaI.0063 and AscI.0060 ApaI.0063. These two PFGE profiles differed by a single slight band shift.

We obtained the whole-genome sequences for two isolates of L. monocytogenes serogroup 1/2a (strains 2015TE19005-1355 and 2015TE24968), representative of the two PFGE clusters and isolated from patients during the outbreak. The complete genomes of these strains were determined with Illumina technology on a NextSeq 500 platform at a coverage of about 90×. Quality control, trimming, and preliminary genome assembly were carried out by an *ad hoc*-implemented pipeline.

The whole-genome sequence of the Listeria monocytogenes 2015TE24968 strain, which assembled in a single circular contig (length, 2,894,716 bp) and is representative of the cluster (PFGE pattern AscI.0183 ApaI.0063) that gathered more than the 80% of all notified human listeriosis cases in that period in central Italy, was submitted to GenBank and annotated using the PGAP (https://www.ncbi.nlm.nih.gov/genomes/static/Pipeline.html). Annotation returned 2,895 genes, 2,795 coding sequences, 6 full rRNA operons, 11 pseudogenes (8 of them were frameshifted genes), 2 clustered regularly interspaced short palindromic repeat (CRISPR) arrays, 4 noncoding RNAs (ncRNAs), and 67 tRNAs.

The cluster of Listeria monocytogenes strains sharing the PFGE AscI.0060 ApaI.0063 pattern did not account for clinical cases since March 2015. The 2015TE19005-1355 strain was chosen as a representative, and its genome was fully sequenced. Analogously, the full-genome sequence assembled in a single circular contig (length, 2,864,291 bp) was submitted to GenBank and annotated by the PGAP. Annotation returned 2,855 genes, 2,757 coding sequences, 6 full rRNA operons, 9 pseudogenes (5 of them were frameshifted genes), 2 CRISPR arrays, 4 ncRNAs, and 67 tRNAs.

Whole-genome alignment between the two genomes highlighted that major differences reside in a large insertion in the 2015TE24968 strain genome of about 31 kb potentially coding for an integrated prophage, which might account for the PFGE band shift.

Moreover, a full-length plasmid (length, 57,530 bp) was detected in both strains. It codes for 62 genes and 2 pseudogenes, and it was found in both clinical and environmental isolates included in the outbreak. Genome sequence availability can help in clarifying evolutionary mechanisms and the genetic relationships among circulating Listeria strains and source trace back.

### Accession number(s).

The genome sequence of the Listeria monocytogenes serotype 1/2a 2015TE24968 strain was deposited at GenBank under the accession number CP014790. The genome sequence of the Listeria monocytogenes serotype 1/2a 2015TE19005-1355 strain was deposited at GenBank under the accession number CP014261. The sequence of the plasmid shared by the two isolates was deposited at GenBank under the accession number CP015985.
